# Account of Diseases in an Eastern District of London, from the 20th of October to the 20th of November, 1800

**Published:** 1801-01

**Authors:** 


*Account of Diſeaſes in an Eaſtern Diſtrict of London, from the 20th of October to the 20th of November, 1800.*

| | |
|---|---|
| ACUTE DISEASES. | |
| Typhus | 25 |
| Peripneumonia Notha | 8 |
| Dyſentery | 7 |
| Cynanche Tonſillaris | 5 |
| CHRONIC DISEASES. | |
| Cough | 25 |
| Dyſpnœa | 10 |
| Cough with Dyſpnœa | 12 |
| Hydrothorax | 5 |
| Hæmoptyſis | 3 |
| Pleurodyne | 2 |
| Diarrhœa | 15 |
| Enterodynia | 5 |
| Chloroſis | 4 |
| Amenorrhœa | 12 |
| Hyſteria | 3 |
| Hypochondriaſis | 7 |
| Hemiplegia | 2 |
| Cephalalgia | 7 |
| Vertigo | 5 |
| Epilepſy | 3 |
| Herpes | 14 |
| Lumbago | 2 |
| Chronic Rheumatiſm | 15 |
| PUERPERAL DISEASES. | |
| Low Puerperal Fever | 5 |
| Abſceſſus Mammæ | 1 |
| Rhagas Papillæ | 3 |
| Iſchuria Veſicalis | 1 |
| INFANTILE DISEASES. | |
| Dentition | 3 |
| Diarrhœa | 7 |
| Convulſio | 2 |

The preſent reigning epidemic is a fever of the typhoid kind, which has proved highly alarming in its ſymptoms, and frequently fatal in its termination. Several medical practitioners, by an aſſiduous attention to the ſick, have become infected by it; and whilſt attempting attempting to relieve the diftreffes of others, have fallen victims to the difeafe. In fome inftances this fever has proceeded very rapidly to a fatal crifis, whilft in others its progrefs has been more flow. It has generally commenced with chillinefs and laffitude, to which have fucceeded an increafed degree of heat, quicknefs of pulfe, and great proftration of ftrength. Giddinefs has very frequently occurred, and this has been the firft fymptom which has occafioned any alarm to the patient or his friends. This fever has generally been attended with great determination to the head, indicated by a flufhed countenance, ferrety eye, a confiderable degree of pain, and in fome inftances by very early delirium. In one of the cafes that occurred to the reporter, the delirium did not occur till a late period, when it proved to be of the moft active and furious kind, and rendered abortive every attempt to remove the difeafe.

Several inftances have occurred of that fpecies of fore throat which has been denominated Cynanche Tonfillaris. In this difeafe, befide other febrile fymptoms, there is an inflammation of different parts of the fauces, but particularly of the tonfils and uvula. Thefe parts become red and tumid, by which a painful deglutition is occafioned, fometimes accompanied with a pain fhooting to the ear. In one of the cafes referred to, there was an unufually large fecretion of mucus, the difcharge of which from the mouth occafioned confiderable fatigue and inconvenience to the patient. By purfuing the antiphlogiftic plan, with moderately aftringent applications to the inflamed parts, and the application of rubefacients to the neck, the difeafe was gradually fubdued.

---

*Account of Difeafes in an Eaftern Diftrict of London, from the 20th of November to the 20th of December,* 1800.

